# Retraction Note: Species-specific chemosensory gene expression in the olfactory organs of the malaria vector *Anopheles gambiae*

**DOI:** 10.1186/s12864-015-1802-z

**Published:** 2015-08-03

**Authors:** Theresa K Hodges, Luciano V Cosme, Giridhar Athrey, Sharmila Pathikonda, Willem Takken, Michel A Slotman

**Affiliations:** Department of Entomology, Texas A&M University, College Station, TX 77843 USA; Laboratory of Entomology, Wageningen University, Wageningen, The Netherlands

## Retraction

The authors would like to retract the article "Species-specific chemosensory gene expression in the olfactory organs of the malaria vector *Anopheles gambiae*" [1]. After the article was published a concern was raised [2] that the expression of a considerable number of olfaction genes we reported for the maxillary palps of *Anopheles gambiae* and *Anopheles quadriannulatus* could be explained by contamination of the maxillary palp sample with antennae. We obtained new gene expression data for both the antennae and maxillary palps for *An. gambiae* and *An. Quadriannulatus*. For the antennae, the new data is essentially the same and the conclusions, particularly with respect to the olfaction genes, remain valid. The maxillary palp data however is considerably different from our previous results in both species and is in line with the previous observations of olfaction gene expression in *An. gambiae* palps [3]. Therefore, we conclude that our previously reported palp data were compromised due to an honest error. We have not been able to determine the cause of the discrepancy, but we cannot rule out tissue cross-contamination. The data will be corrected and a new manuscript will be submitted for peer review. We apologise for any inconvenience caused.
